# The Role of Circulating Tumor DNA in Advanced Non-Small Cell Lung Cancer Patients Treated With Immune Checkpoint Inhibitors: A Systematic Review and Meta-Analysis

**DOI:** 10.3389/fonc.2021.671874

**Published:** 2021-07-21

**Authors:** Haowei Wang, Fei Zhou, Meng Qiao, Xuefei Li, Chao Zhao, Lei Cheng, Xiaoxia Chen, Caicun Zhou

**Affiliations:** ^1^ Department of Medical Oncology, Shanghai Pulmonary Hospital, School of Medicine, Tongji University, Shanghai, China; ^2^ Department of Lung Cancer and Immunology, Shanghai Pulmonary Hospital, School of Medicine, Tongji University, Shanghai, China

**Keywords:** non-small-cell lung cancer, immune checkpoint inhibitor, circulating tumor DNA, biomarker, survival

## Abstract

**Background:**

The use of circulating tumor DNA (ctDNA) to reflect clinical benefits of advanced non-small cell lung cancer (NSCLC) patients during immune checkpoint inhibitor (ICI) therapy remains controversial. This study aimed to determine the association of pre-treatment and early dynamic changes of ctDNA with clinical outcomes in advanced NSCLC patients treated with ICIs.

**Methods:**

Electronic databases (PubMed, Embase, Web of Science, and Cochrane) were systematically searched to include relevant studies published in English up to November 2020. The primary outcomes were overall survival (OS) and progression-free survival (PFS) and the secondary outcome was objective response rate (ORR) with RECIST criteria.

**Results:**

A total of 1017 patients from 10 studies were identified. The baseline ctDNA levels (detected *versus* not detected) showed no significant association with clinical outcomes regarding OS (hazard ratio [HR], 1.18; 95% confidence interval [CI], 0.93-1.51), PFS (HR, 0.98; 95% CI, 0.80-1.21), and ORR (odds ratio [OR], 0.89; 95% CI, 0.54-1.46). Interestingly, when taken early longitudinal assessment of ctDNA into consideration, the early reduction of the concentration of ctDNA was associated with significant improvements of OS (HR, 0.19; 95% CI, 0.10-0.35), PFS (HR, 0.30; 95% CI, 0.22-0.41) and ORR (OR, 0.07; 95% CI, 0.03-0.18). Further subgroup analyses revealed that the reduction magnitude did not significantly impact the association between ctDNA and clinical outcomes, suggesting that both patients with decreased ctDNA or a ≥50% reduction of ctDNA was associated with improved OS, PFS and ORR.

**Conclusion:**

Early reduction of ctDNA was associated with improved OS, PFS and ORR in advanced NSCLC patients treated with ICIs.

**Systematic Review Registration:**

https://www.crd.york.ac.uk/PROSPERO, CRD42021226255.

## Introduction

Over the past decade, immunotherapy, targeting immune checkpoint molecules, programmed-death-1(PD-1)/PD ligand-1 (PD-L1) axis, has turned into one of the most important breakthroughs in cancer treatment, including non-small cell lung cancer (NSCLC) (1). Although treatment of NSCLC with immune checkpoint inhibitors (ICIs) can produce remarkably durable responses, a considerable proportion of patients cannot derive meaningful benefits from ICI therapy owing to drug resistance (2), disease hyper-progression (3), or immune-related adverse events (irAEs) (4).

Currently, several promising biomarkers for ICI therapy have been widely investigated, such as tumor mutation burden (TMB), PD-L1 expression, germline genotype of HLA-I, the molecular profiling of the tumor microenvironment, mutations in DNA mismatch repair and replication genes (1, 5, 6). However, most of these biomarkers are far from perfect biomarkers owing to being invasive, not always feasible, and its spatial and temporal heterogeneity (6, 7).

In response to the demand for genetic predictive and non-invasive molecular biomarkers in NSCLC, liquid biopsy, including circulating tumor DNA (ctDNA), have been developed. ctDNA, referring to a sub-set of cell-free DNA, is released by tumor cells undergoing apoptosis, necrosis, and in extracellular vesicles (exosomes) secreted from tumor cells and can be found in plasma. ctDNA is highly fragmented and ranges between 100 and 200 base pairs in size and represents genetic material from the primary tumor as well as metastases (8). ctDNA can be quantified using multiple metrics, such as mutant allele fraction or mutant allele concentration (that is, copies per milliliter) (9). The level of ctDNA in plasma has been demonstrated to correlate with tumor size (10, 11), disease stage (12), the clinical responses and prognosis of patients receiving anti-tumor treatment (13–16). The short half-life of ctDNA (17, 18), as well as the reduced and ease risk of repeating liquid biopsies relative to tissue biopsies (19) or imaging (20), enables ctDNA to be used for real-time monitoring of tumor burden in response to treatment. Although NSCLC is the cancer type for which plasma ctDNA testing has the most comprehensive and compelling evidence (21), it is still controversial regarding the role of ctDNA in predicting survival and clinical response.

ctDNA assays for the evaluation of cancers that harbor *EGFR*-sensitizing or *EGFR*-resistance mutations have already entered into clinical practice (22). A recent prospective study using ctDNA to guide matched targeted therapy in lung cancers supported the incorporation of plasma ctDNA into clinical practice (23). In addition to the direction of molecular targeted treatment, ctDNA could also potentially help monitor ICI response as the quantitative level in plasma has been demonstrated to reflect the tumor burden in patients even earlier than clinical detection (17, 24–27) and might identify response earlier than clinical detection (28, 29). However, these studies are mostly retrospective design, lacking high-level medical evidence, and some results were even inconsistent. Therefore, we conducted this meta-analysis to comprehensively investigate the predictive value of ctDNA for advanced NSCLC patients who received ICI therapy.

## Method

The authors declare that all supporting data, study materials and analytic methods within the article and the online supporting information are available to other researchers. This systematic review was performed in adherence to the Preferred Reporting Items for Systematic Reviews and Meta-Analyses (PRISMA) guidelines (30). The PRISMA checklist is provided in **Supplementary Table S1**.

### Search Strategy

The systematic search of the scientific literature was performed. The search was conducted up to November 2020 in PubMed, EMBASE, Web of Science, and the Cochrane Library database. The main keywords used for the online search were “Circulating Tumor DNA,” “Carcinoma, Non-Small Cell Lung,” “Immune Checkpoint Inhibitor.” The full online search strategies were demonstrated in **Supplementary Text S1**. We also manually examined the references of each screened study until no additional articles could be added.

### Exclusion and Inclusion Criteria

Studies were selected if they met the following inclusion criteria (1): patients with advanced NSCLC; (2) patients received ICIs alone or ICI-based therapy; (3) ctDNA was analyzed in these groups; (4) information on the clinical response or prognosis of these patients were provided; (5) if multiple studies from the same populations were available, to avoid repetition information, only the one with the largest sample size was included. Reviews, case reports, conference reports, abstracts, phase I studies and non-English publications were excluded. Endnote (Thomson Research Soft; Stanford, Connecticut, the United States) was used to select and screen the literature.

### Data Extraction

Data extraction and analyses were performed by 2 independent reviewers (HW and FZ). Any disagreement was discussed with the senior author (XC). Extracted study characteristics included: first author, publication year, country of the study, number of patients who underwent ICIs alone or combined therapy, gender, median/mean age, histological types, stage, smoking history, PD-L1 expression, ECOG PS, follow up duration, drugs, sample, extraction method, detection method, platform, detection time, most mutated genes.

### Outcomes

The primary outcomes were overall survival (OS) and progression-free survival (PFS), then the secondary outcome included objective response rate (ORR) with RECIST criteria.

### Risk of Bias Assessment

To assesses the quality of non-randomized studies (31), the Newcastle-Ottawa scale was used to assess the risk of bias. The scale assesses risk of bias in the following 3 aspects: selection of the study which include adequate definition and representation of the case, comparability of groups, and ascertainment of exposure and outcome for cases and controls. Studies with score less than 4 were considered as a high risk of bias, those with scores of 4 to 6 were regarded to have an intermediate risk of bias, and scores of 7 or more represented a low risk of bias. The results displayed in **Supplementary Table S2**

### Statistical Analysis

The heterogeneity of different studies was tested by using the Q test. The HRs and ORs with 95% CIs were directly extracted from the research article or calculated using previously published methods, as proposed by Tierney et al. (32). We calculated the I^2^ to assess the extent of variability attributable to statistical heterogeneity across studies. I^2^ < 50% and P > 0.10 were interpreted as signifying low-level heterogeneity. Across the studies, if no significant heterogeneity was found, the results were combined with the fixed-effects model (Mantel–Haenszel) (33); otherwise, the random-effects model (DerSimonian-Laird) was used (34). Publication bias was assessed by visual inspection of a funnel plot, Begg’s and Egger’s tests. A sensitivity analysis was performed by serially excluding each study to determine its influence. P values were two-sided and considered significant if less than 0.05. STATA 15.1 software for Mac was used to evaluate the outcomes.

### Subgroup Analysis

The following subgroup analyses were performed. Firstly, as already assessed, the patients were stratified with two groups depending on different cutoff value of longitudinal ctDNA dynamic. Then, we also performed subgroup analysis considering that different platforms in studies might provide heterogeneity. The details of groups and outcomes were provided in the results of subgroup analysis.

## Results

### Study Selection

**Figure 1:**
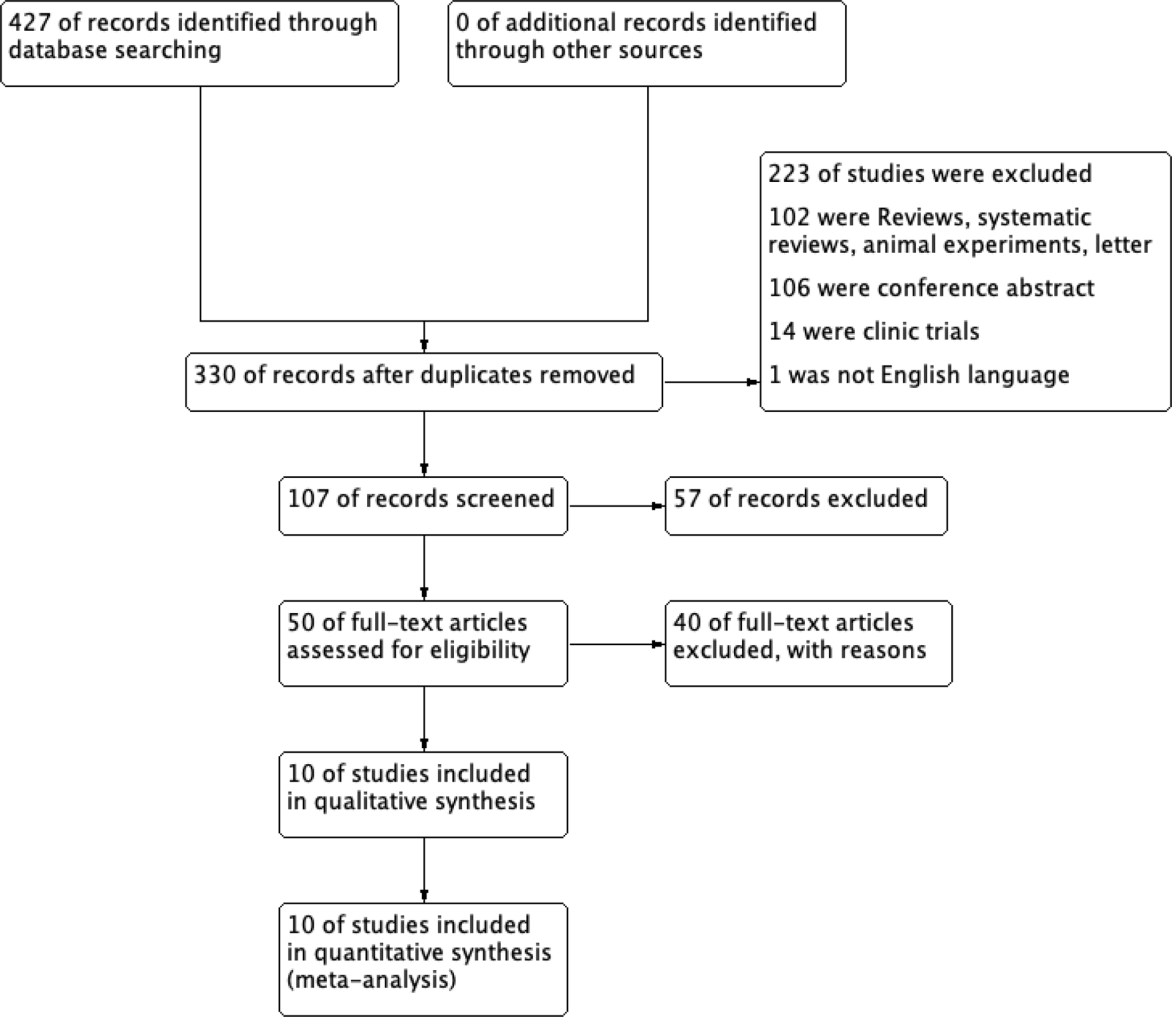
Flow diagram of the study selection.

Of the 427 articles retrieved by the literature search, 97 duplicates were removed, left 330 studies available for screening. 50 studies underwent full-text review, after screening the title and abstract. Of these studies,10 pieces of literature met the inclusion criteria and were chose for the current meta-analysis (7, 35–43). The detailed flow chart is shown in **Figure 1**.

### Study Characteristics

The basic characteristics of the studies are summarized in **Table 1**. A total of 1017 patients were included in the study. Data from 3 studies (7, 36, 43) analyzed the PFS, 3 studies (36, 37, 43) considered OS and 4 studies (35, 36, 39, 43) discussed the ORR with the association of baseline ctDNA. 7 studies (37–43) presented PFS, 4 studies (37–39, 43) reported OS and 6 (35, 37–39, 41, 43) studies treated ORR as one of the results in early dynamic assessment of ctDNA. There were 2 studies (36, 38), both of which had 2 independent cohorts, so we used (1) and (2) to distinguish them.

**Table 1:** Basic characteristics of the included studies in the present meta-analysis.

First author	Year	Country	Total cases	Female(%)	Age	Histological types			Stage		Smoking history			PD-L1 Expression			ECOG PS		Follow up duration median or up to(M)
						NSCC		SCC	III	IV	current	former	never	positive	negative	unknown	0	≥1	
Iijima, Y.	2017	Japan	14.00	5 (36)	66	10		4	0	14	0	10	4	2	0	12	10	4	10
Gandara, D. R (1)	2018	US	144.00	51 (35)	62	95		49	advanced		25	92	27	NA	NA	NA	48	96	27
Gandara, D. R (2)			425.00	164 (39)	63	313		112	advanced		59	282	84	NA	NA	NA	155	270	27
Goldberg, S. B	2018	US	49.00	31 (63)	67	47		2	advanced		1	43	5	NA	NA	NA	NA	NA	20
Raja, R (1)	2018	US	28.00	8 (29)	62	10		18	3	25	22		6	13	13	2	10	18	15
Raja, R (2)			72.00	30 (42)	61	57		15	16	56	59		13	58	11	3	27	45	9
Anagnostou, V.	2019	US	24.00	12 (50)	64	16		8	0	24	3	18	3	NA	NA	NA	NA	NA	12.7
Guibert, N	2019	French	97.00	37 (38)	NA	76		21	11	86	22	63	7	35	40	22	87(0-2)	4(≥2)	24
Chen, Y.	2020	China	22.00	5 (23)	62	10		12	5	17	15		7	11	4	7	NA	NA	15
Jia, Q	2020	China	9.00	1 (11)	65	6		3	0	9	0	8	1	NA	NA	NA	NA	NA	10
Nabet, B. Y.	2020	US	99.00	51 (52)	65	85		14	advanced		13	64	22	58	24	17	NA	NA	50
Zulato, E	2020	Italy	34.00	NA	68	NA		NA	advanced		NA	NA	NA	NA	NA	NA	NA	NA	13.1
**First author**	**Year**	**Drugs (number)**				**Previous therapy lines**			**Sample**		**Detection method**			**Platform**			**Detection time (weeks)**		**Most mutated genes**
						**0-1**	**>1**												
Iijima, Y.	2017	Nivolumab (14)				NA			plasma		NGS			Ion Proton			baseline, 1, 2, 4, 6,8		TP53
Gandara, D. R (1)	2018	Atezolizumab (144)				93	51		plasma		NGS			Illumina HiSeq 4000			baseline		KRAS
Gandara, D. R (2)		Atezolizumab (425)				320	105		plasma		NGS			Illumina HiSeq 4000			baseline		EGFR
Goldberg, S. B	2018	anti–PD-1 (36); anti–PD-L1 (2) anti-PD-L1+ID01 inhibitor (2) anti-CTLA-4+anti PD-L1 (7) anti-CTLA-4+anti PD-1 (2)				40	9		plasma		NGS			Illumina HiSeq 2500			baseline, 2		KRAS
Raja, R (1)	2018	Durvalumab (28)				11	17		plasma		NGS			Guardant360			baseline, 6		TP53
Raja, R (2)		Durvalumab (72)				0	72		plasma		NGS			Guardant360			baseline, 6		TP53
Anagnostou, V.	2019	Nivolumab (14); Pembrolizumab (5) Nivolumab+anti-LAG3 (1) Nivolumab+Ipilimumab (1) Pembrolizumab+chemotherapy (3)				NA			plasma		NGS			Illumina HiSeq 2500			baseline, 4 or 8, the time of disease progression		KRAS
Guibert, N	2019	Nivolumab (90); Pembrolizumab (7)				57	40		plasma		NGS			Illumina NextSeq 500			baseline, 4		KRAS
Chen, Y.	2020	Camrelizumab + Apatinib (22)				14	8		plasma		NGS			Illumina Novaseq 6000			baseline		TP53
Jia, Q	2020	Durvalumab (4) Tremelimumab+Durvalumab (5)				NA			plasma		NGS			Illumina Novaseq 6000			baseline, 8		TTN
Nabet, B. Y.	2020	anti–PD-L1 (1); anti–PD-1 (31) anti–PD-1+anti–CTLA-4 (5) anti–PD-1+Chemotherapy (5)				41	58		plasma		NGS			Illumina HiSeq4000			baseline, the time of the second infusion		TP53
Zulato, E	2020	Nivolumab (12) Pembrolizumab (18) Atezolizumab (4)				NA			plasma		ddPCR			Bio-Rad QX200			baseline, first radiological restaging		KRAS

NSCC, Non-squamous cell carcinoma; SCC, Squamous cell carcinoma; NA, not applicable; NGS, Next-generation sequencing; ddPCR, Droplet Digital PCR; M, months.

### The Association of Baseline (Pre-ICI Therapy) ctDNA and Clinical Outcomes

**Figure 2:**
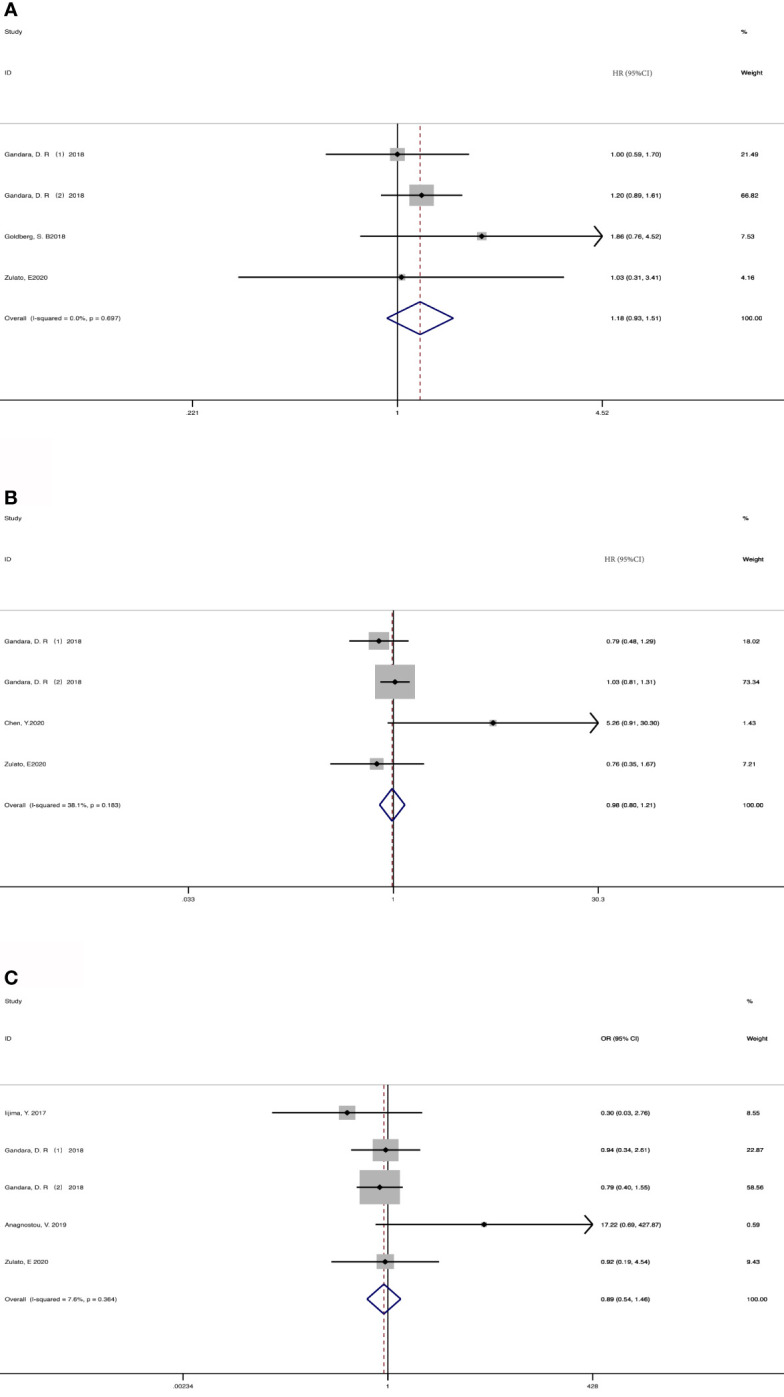
Meta-analysis of the associations between baseline ctDNA and **(A)** overall survival, **(B)** progression-free survival, **(C)** objective response rate. HR, hazard ratio; OR, odds ratio.

4 cohorts from 3 studies (36, 37, 43) were included for analyzing the association between baseline ctDNA and OS (**Figure 2A**). Considering that the results were not heterogeneous, we chose fixed effects models. There was no statistical significance between the two groups regarding OS (HR, 1.18; 95% CI, 0.93-1.51; I^2^<0.1%; p=0.181). Data from 3 studies (7, 36, 43) and 4 cohorts were pooled for the analysis of PFS (**Figure 2B**), with a total of 625 patients. Considering that the results were homogeneous, we also used fixed-effects models. There was still no evident difference between the groups in terms of PFS (HR,0.98; 95% CI, 0.80-1.21; I^2 =^ 38.1%; p=0.865) The results of 4 studies (35, 36, 39, 43) and 5 cohorts were pooled to examine the relationship of baseline detected ctDNA and ORR (**Figure 2C**), with a sample size of 641 patients. The pooled OR in the detected group with ORR (OR,0.89; 95% CI,0.54-1.46; I^2 =^ 7.6%; p=0.641) showed no significant difference between the two groups.

### The Association of Early Reduction of ctDNA and Clinical Outcomes

**Figure 3:**
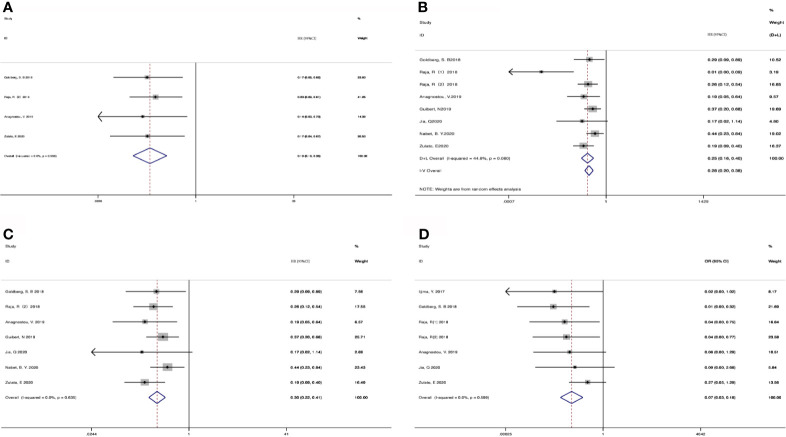
Meta-analysis of the associations between early reduction of ctDNA and **(A)** overall survival, **(B, C)** progression-free survival, **(D)** objective response rate.

**Figure 4:**
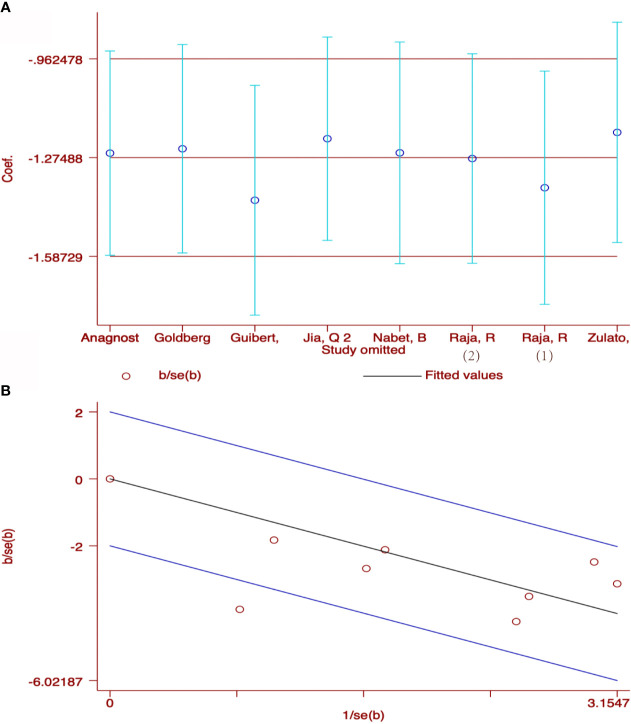
Sensitivity analysis **(A)** and Star Plot **(B)** of the included literatures on the reduction of ctDNA with PFS as the result.

207 patients from 5 cohorts in 4 studies (37–39, 43) were included to investigate the pooled association between early reduction of ctDNA and OS. Eight cohorts from 7 studies (37–43) were included to survey the relation between early assessment of ctDNA and PFS, with a sample size of 412 patients. The pooled HR for OS with the fixed-effects model (**Figure 3A**) was 0.19 (95%CI, 0.10-0.35; I^2^<0.1%; p<0.001); the HR for PFS with random-effects model (**Figure 3B**) was 0.25 (95%CI, 0.16-0.40; I^2^ = 44.8%; p<0.001); Sensitivity analysis and Star Plot were performed because of the significant heterogeneity (**Figure 4**). When excluding group 1 in the study by Raja et al. (38), the heterogeneity decreased to I-squared<0.1%; with a pooled HR (**Figure 3C**) of 0.30 (95%CI, 0.22-0.41; p<0.001). In the early reduction ctDNA group, the pooled OR (**Figure 3D**) for ORR in 6 studies (35, 37–39, 41, 43) with 230 patients with fixed-effect models was 0.07 (95% CI,0.03-0.18; I^2^<0.1%; p<0.001).

### Subgroup Analysis in Longitudinal Observation of Patients With ICIs Therapy and Platform

**Figure 5:**
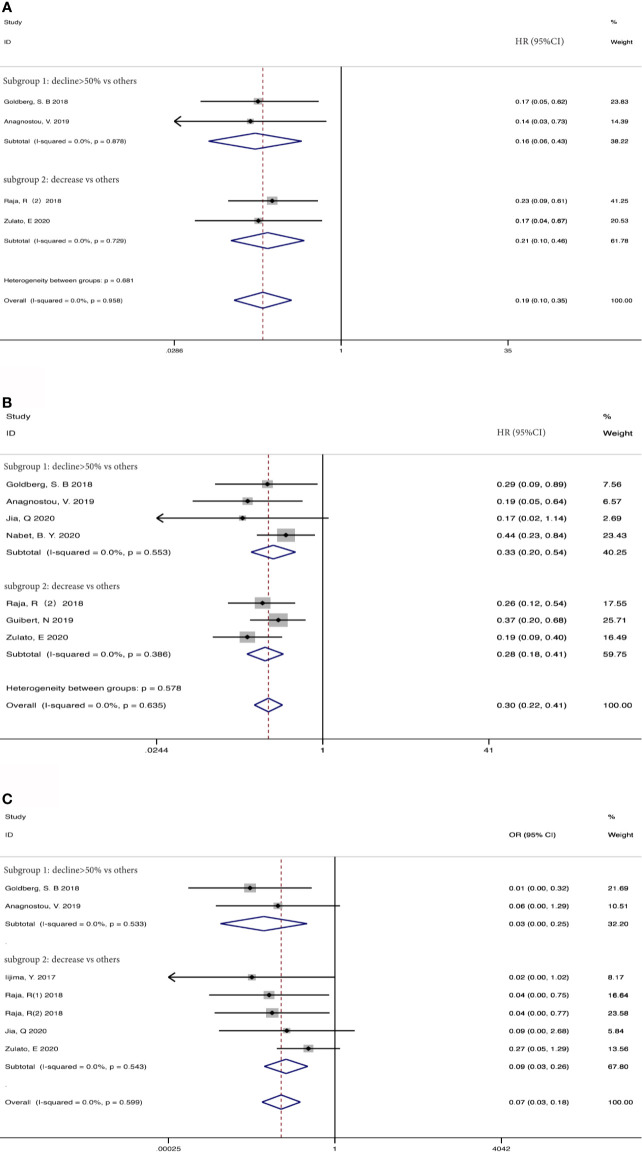
Subgroup analysis the associations between early decrease of ctDNA and **(A)** overall survival, **(B)** progression-free survival, **(C)** objective response rate.

Taking into account these studies, the authors selected different degrees of the reduction of ctDNA during treatment as the cutoff to define the positive group. Therefore, we further adopted subgroup analysis to divide the studies that dropped into the positive group and those with different degrees of decline into two subgroups: a decline of >50% as the threshold to define ctDNA response (subgroup 1) *vs* others; decreased *vs* others (subgroup 2). 73 patients from two studies (37, 39) were included in subgroup 1 with a HR (**Figure 5A**) of 0.16 for OS (95%CI, 0.06-0.43; I^2^ <0.1%, p<0.001), 106 patients from two studies were included in subgroup 2 with a HR (**Figure 5A**) of 0.21 for OS (95%CI, 0.10-0.46; I^2^ <0.1%, p<0.001). 181 patients from 4 studies (37, 39, 41, 42) were included in subgroup 1 with a HR (**Figure 5B**) of 0.33 for PFS (95%CI, 0.20-0.54; I^2^ <0.1%, p<0.001). 203 patients from 3 studies (38, 40, 43) were included in subgroup 2 with a HR (**Figure 5B**) of 0.28 for PFS (95% CI, 0.18-0.41; I^2^ <0.1%, p<0.001). When regarding ORR, 73 patients from 2 studies (37, 39) were included in subgroup 1 with an OR (**Figure 5C**) of 0.03 (95%CI,0.01-0.25; I^2^<0.1%, p=0.001), and 157 patients from 4 studies (35, 38, 41, 43) in subgroup 2 with an OR (**Figure 5C**) of 0.09 (95%CI,0.03-0.26 I^2^ <0.1%, p<0.001). Considering that most studies included used illumina platform, the subgroup was utilized to analyze the difference of illumina platform *vs* other platforms (**Supplement Figure S1**). The results still maintained coherence in different platforms both in baseline detection and dynamic observation, showing consistent with previous research (44).

### Publication Bias

**Figure 6:**
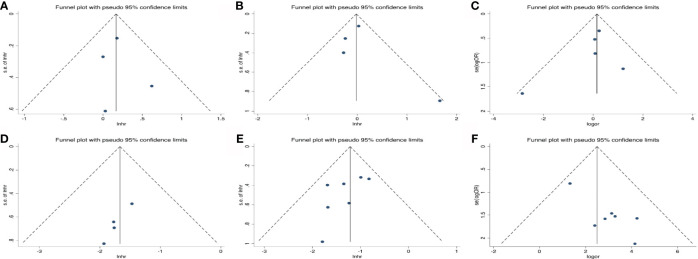
Publication bias for the association of detected baseline ctDNA with **(A)** overall survival, **(B)** progression-free survival, **(C)** objective response rate and **(D)** the relationship of early decreased ctDNA with OS; **(E)** PFS; **(F)** ORR.

As shown in **Figure 6**, the funnel plots were almost symmetrical and the test results indicated that no publication bias existed regarding the HRs for OS (Begg’s test, p=1.000; Egger’s test, p=0.802), PFS (Begg’s test, p=0.734; Egger’s test, p=0.699) and OR for ORR (Begg’s test, p= 0.806; Egger’s test, p=0.493) in baseline detected ctDNA. However, there are inconsistent results when taking early ctDNA dynamics into consideration, with HRs for OS (Begg’s test, p=0.308; Egger’s test, p=0.023) or PFS (Begg’s test, p=0.230; Egger’s test, p=0.160) and OR for ORR (Begg’s test, p= 0.548; Egger’s test, p=0.009), suggesting that publication bias might exist among these studies.

## Discussion

To our knowledge, this was the first meta-analysis to comprehensively investigate predictive significance of ctDNA in advanced NSCLC patients treated with ICI therapy. The current study found that early reduction of ctDNA was associated with improved PFS, OS and ORR while there was no significant association between baseline ctDNA and clinical outcomes.

The concept of monitoring tumor burden and therefore biological effects of treatment by analyzing circulating biomarkers has been known for long, but only recently the availability of techniques able to detect ctDNA has opened new perspectives (17, 45). In lung cancer, the first experience concern *EGFR*-mutated disease (46). The use of ctDNA as a quantitative biomarker for assessment of ICI response has been investigated in prior studies (47–50), most of which have focused on tracking driver mutations in patients using digital PCR or allele-specific PCR. Even previous studies concluded that pretreatment ctDNA level appears to be an independent, inversely prognostic variable across tumor types, characterized by an association with OS and other known prognostic variables, but not with ORR (51). In this meta-analysis, we did not find a significant association between baseline ctDNA and clinical outcomes. Compared with tumor tissue, the abundance of tumor DNA in plasma samples is relatively low, which poses a great challenge to the sensitivity of plasma detection. False-negative results may occur due to insufficient plasma ctDNA content or lacking of inclusion of altered genes in the targeted NGS panel (21). On the other hand, false-positive results may also occur because of germline variants or the presence of somatic mutations in hematopoietic stem cells owing to clonal hematopoiesis, although most plasma ctDNA assays use matched sequencing of white blood cells (52–54), there still be sequencing errors and artifacts (55). A recent study found that high allele frequency blood TMB was strongly correlated with the ctDNA amount (56). However, high allele frequency blood TMB was a negative prognostic factor rather than a predictive factor (56), this may partially explain that there was no significant association between baseline ctDNA and clinical outcomes.

Interestingly, in this meta-analysis, patients with the reduced dynamic ctDNA obviously obtained more clinical benefits from ICI therapy. The early reduction of ctDNA may reflect an early response of tumors to effective treatment. This was consistent with previous studies, the amount of ctDNA may be an independent factor to predict the efficacy of patients receiving ICIs, and combined with other predictive indicators can better distinguish potential benefit populations (42). Subgroup analysis also indicated that a decline of >50% as a threshold to define ctDNA response and even achievement of undetectable ctDNA may prove to be a stronger predictor of long-term response, compared with just defining ctDNA response as decrease during ICIs therapy, and may identify patients who comprise the “tail” of the survival curve (57). Nevertheless, it still needs prospective studies to confirm these findings. Interestingly, some studies report a transient spike preceding a decline in ctDNA levels in a subset of patients, likely reflecting DNA release as tumor cells are killed. It would be important to avoid misinterpreting such a spike as disease progression (37, 58, 59).

There still were some limitations in our analysis. Firstly, all data were extracted from retrospective or post-hoc analysis studies. Secondly, the quality of data was heterogeneous as several pieces of important information such as prior therapy were not consistently reported. Finally, a high proportion of patients in these studies had *KRAS* and *TP53* mutations. As *TP53* and *KRAS* mutations showed remarkable clinical benefits from ICIs (60), it remains undermined that whether detecting TP53 and KRAS in ctDNA for predicting the response to ICIs was as effective as total ctDNA.

In summary, this current study suggested that early reduction of ctDNA was associated with improved PFS, OS and ORR in advanced NSCLC patients treated with ICIs. Further large-scale and rigorously designed prospective studies are still warranted to verify its clinical value. In the future, clinical trials will be conducted in NSCLC patients to determine whether ctDNA could be selected as a significant factor to monitor clinical response to ICI therapy.

## Data Availability Statement

The original contributions presented in the study are included in the article/**Supplementary Material**. Further inquiries can be directed to the corresponding authors.

## Author Contributions

HW and FZ collected the relevant papers and data and drafted the manuscript text. HW, MQ and XL performed statistical analyses. HW, CZ, and LC performed the quality assessment. XC and CCZ gave critical comments and revised the paper. All authors contributed to the article and approved the submitted version.

## Funding

This study was supported in part by grants from the Shanghai “Rising Stars of Medical Talent” Youth Development Program (to FZ) National Natural Science Foundation of China (No. 81703020), Medical Guidance Project of Shanghai Science and Technology Commission (No. 17411969200), and Shanghai Innovative Collaboration Project (No. 2020CXJQ02).

## Conflict of Interest

The authors declare that the research was conducted in the absence of any commercial or financial relationships that could be construed as a potential conflict of interest.

## Supplementary Material

Supplementary Figure 1 Subgroup analysis of the different platforms in baseline detection with **(A)** overall survival, **(B)** progression-free survival, **(C)** objective response rate and in dynamic ctDNA with **(D)** overall survival, **(E)** progression-free survival, **(F)** objective response rate. HR, hazard ratio; OR, odds ratio.
