# Supplementary figures and images for: The Role of Circulating Tumor DNA in Advanced Non-Small Cell Lung Cancer Patients Treated With Immune Checkpoint Inhibitors: A Systematic Review and Meta-Analysis
Source: Front Oncol. 2021 Jul 21;11:671874. doi: 10.3389/fonc.2021.671874 (PMC8335591; doi:10.3389/fonc.2021.671874)

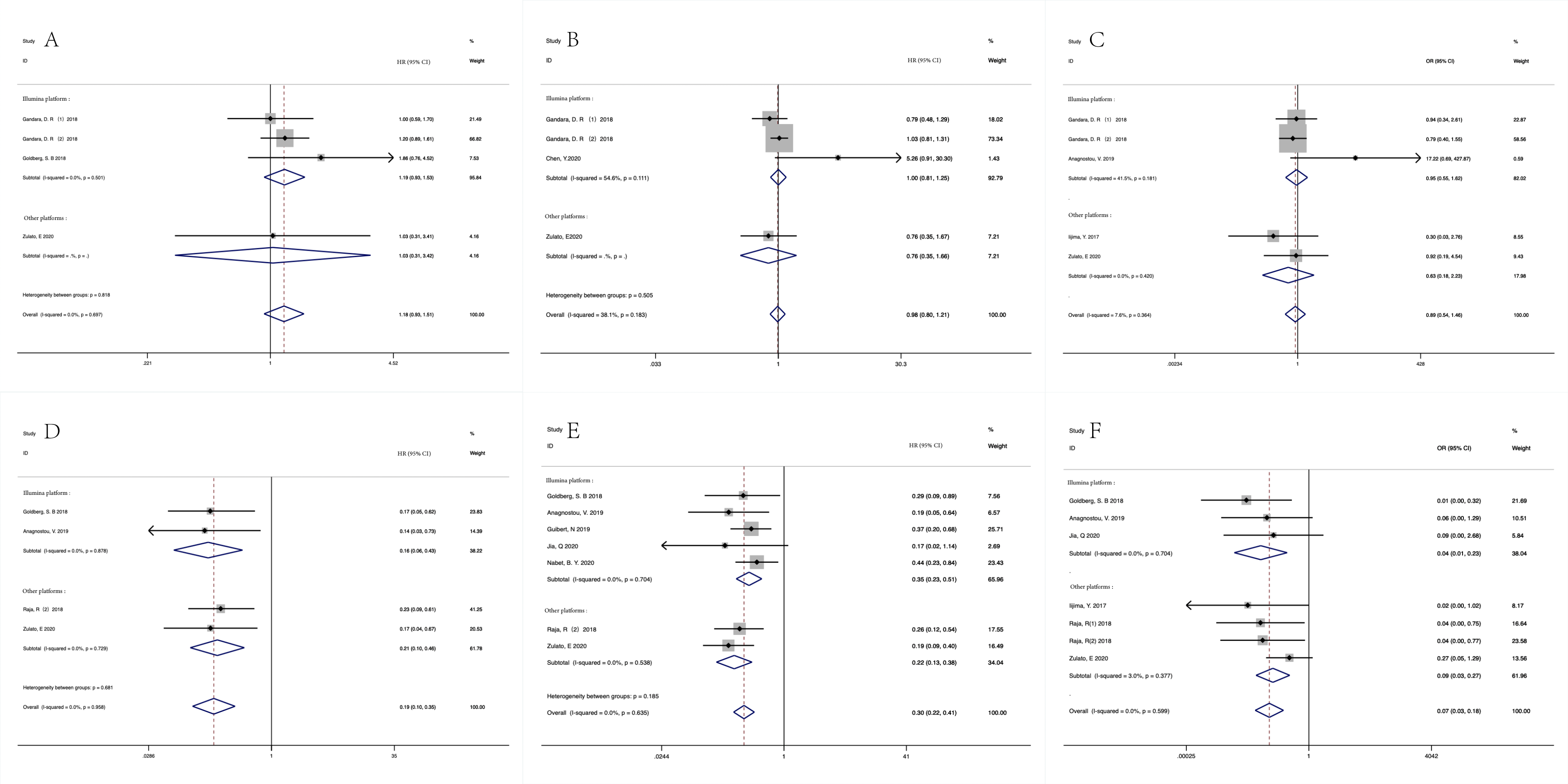

Supplement: Supplementary Figure 1 — Subgroup analysis of the different platforms in baseline detection with (A) overall survival, (B) progression-free survival, (C) objective response rate and in dynamic ctDNA with (D) overall survival, (E) progression-free survival, (F) objective response rate. HR, hazard ratio; OR, odds ratio. [file Image_1.tif]
