# Supplementary material for: The Role of Circulating Tumor DNA in Advanced Non-Small Cell Lung Cancer Patients Treated With Immune Checkpoint Inhibitors: A Systematic Review and Meta-Analysis
Source: Front Oncol. 2021 Jul 21;11:671874. doi: 10.3389/fonc.2021.671874 (PMC8335591; doi:10.3389/fonc.2021.671874)
Supplement: Supplementary file 2 [file DataSheet_1.docx]

**Supplementary** **Text S1．Search Strategies**

Search included: PubMed, EMBASE, Web of Science, and Cochrane: date was from the inception through November 10, 2020**.**

1. **PubMed search strategy**

(((((((((Circulating Tumor DNA[Title/Abstract]) OR (DNA, Circulating Tumor[Title/Abstract])) OR (Tumor DNA, Circulating[Title/Abstract])) OR (Cell-Free Tumor DNA[Title/Abstract])) OR (Cell Free Tumor DNA[Title/Abstract])) OR (DNA, Cell-Free Tumor[Title/Abstract])) OR (Tumor DNA, Cell-Free[Title/Abstract])) OR ("Circulating Tumor DNA"[Mesh])) AND (((((((((((Carcinoma, Non Small Cell Lung[Title/Abstract]) OR (Carcinomas, Non-Small-Cell Lung[Title/Abstract])) OR (Lung Carcinoma, Non-Small-Cell[Title/Abstract])) OR (Lung Carcinomas, Non-Small-Cell[Title/Abstract])) OR (Non-Small-Cell Lung Carcinomas [Title/Abstract])) OR (Nonsmall Cell Lung Cancer[Title/Abstract])) OR (Non-Small-Cell Lung Carcinoma[Title/Abstract])) OR (Non Small Cell Lung Carcinoma[Title/Abstract])) OR (Carcinoma, Non-Small Cell Lung[Title/Abstract])) OR (Non-Small Cell Lung Cancer[Title/Abstract])) OR ("Carcinoma, Non-Small-Cell Lung"[Mesh]))) AND (("Immunotherapy"[Mesh]) OR (((((((((((((((((Immune Checkpoint Inhibitor[Title/Abstract]) OR (immune therapy[Title/Abstract])) OR (Immunotherapy[Title/Abstract])) OR (ipilimumab[Title/Abstract])) OR (Tremelimumab[Title/Abstract])) OR (Nivolumab [Title/Abstract])) OR (Pembrolizumab[Title/Abstract])) OR (Atezolizumab[Title/Abstract])) OR (Durvalumab[Title/Abstract])) OR (Avelumab[Title/Abstract])) OR (cytotoxic T-lymphocyte associated antigen-4[Title/Abstract])) OR (CTLA-4[Title/Abstract])) OR (programmed cell death protein-1[Title/Abstract])) OR (programmed cell death protein [Title/Abstract])) OR (PD-1[Title/Abstract])) OR (programmed cell death-Ligand 1 [Title/Abstract])) OR (PD-L1[Title/Abstract])))

**2) EMBASE search strategy**

("Carcinoma, Non Small Cell Lung":ab,ti or "Carcinomas, Non-Small-Cell Lung":ab,ti or "Lung Carcinoma, Non-Small-Cell":ab,ti or "Lung Carcinomas, Non-Small-Cell":ab,ti or "Non-Small-Cell Lung Carcinomas":ab,ti or "Nonsmall Cell Lung Cancer":ab,ti or "Non-Small-Cell Lung Carcinoma":ab,ti or "Non Small Cell Lung Carcinoma":ab,ti or "Carcinoma, Non-Small Cell Lung":ab,ti or "Non-Small Cell Lung Cancer":ab,ti or 'non small cell lung cancer'/exp) AND ("Circulating Tumor DNA":ab,ti or "DNA, Circulating Tumor":ab,ti or "Tumor DNA, Circulating ":ab,ti or "Cell-Free Tumor DNA":ab,ti or "Cell Free Tumor DNA":ab,ti or "DNA, Cell-Free Tumor":ab,ti or "Tumor DNA, Cell-Free":ab,ti or 'circulating tumor dna'/exp) AND ("Immune Checkpoint Inhibitor":ab,ti or "immune therapy":ab,ti or "Immunotherapy":ab,ti or "ipilimumab":ab,ti or "Tremelimumab":ab,ti or "Nivolumab":ab,ti or "Pembrolizumab ":ab,ti or "Atezolizumab":ab,ti or "Durvalumab":ab,ti or"Avelumab":ab,ti or "cytotoxic T-lymphocyte associated antigen-4":ab,ti or "CTLA-4":ab,ti or "programmed cell death protein-1":ab,ti or "programmed cell death protein":ab,ti or "PD-1":ab,ti or "programmed cell death-Ligand 1":ab,ti or "PD-L1":ab,ti or 'immunotherapy'/exp)

**3) Cochrane database search strategy**

(Circulating Tumor DNA) or (DNA, Circulating Tumor) or (Tumor DNA, Circulating) or (Cell-Free Tumor DNA) or (Cell Free Tumor DNA) or (DNA, Cell-Free Tumor) or (Tumor DNA, Cell-Free) AND (Carcinoma, Non Small Cell Lung) or (Carcinomas, Non-Small-Cell Lung) or (Lung Carcinoma, Non-Small-Cell) or (Lung Carcinomas, Non-Small-Cell) or (Non-Small-Cell Lung Carcinomas) or (Nonsmall Cell Lung Cancer) or (Non-Small-Cell Lung Carcinoma) or (Non Small Cell Lung Carcinoma) or (Carcinoma, Non-Small Cell Lung) or (Non-Small Cell Lung Cancer) AND (Immune Checkpoint Inhibitor) or (immune therapy) or (Immunotherapy) or (ipilimumab) or (Tremelimumab) or (Nivolumab) or (Pembrolizumab) or (Atezolizumab) or (Durvalumab) or (Avelumab) or (cytotoxic T-lymphocyte associated antigen-4) or (CTLA-4) or (programmed cell death protein-1) or (programmed cell death protein) or (PD-1) or (programmed cell death-Ligand 1) or (PD-L1)

**4) Web of Science search strategy**

(Circulating Tumor DNA OR DNA, Circulating Tumor OR Tumor DNA, Circulating OR Cell-Free Tumor DNA OR Cell Free Tumor DNA OR DNA, Cell-Free Tumor OR Tumor DNA, Cell-Free) AND (Carcinoma, Non-Small-Cell Lung OR Carcinoma, Non Small Cell Lung OR Carcinomas, Non-Small-Cell Lung OR Lung Carcinoma, Non-Small-Cell OR Lung Carcinomas, Non-Small-Cell OR Non-Small-Cell Lung Carcinomas OR Nonsmall Cell Lung Cancer OR Non-Small-Cell Lung Carcinoma OR Non Small Cell Lung Carcinoma OR Carcinoma, Non-Small Cell Lung OR Non-Small Cell Lung Cancer) AND(Immune Checkpoint Inhibitor OR immune therapy OR Immunotherapy OR ipilimumab OR Tremelimumab OR Nivolumab OR Pembrolizumab OR Atezolizumab OR Durvalumab OR Avelumab OR cytotoxic T-lymphocyte associated antigen-4 OR CTLA-4 OR programmed cell death protein-1 OR programmed cell death protein OR PD-1 OR programmed cell death-Ligand 1 OR PD-L1)

**Table S1. PRISMA checklist.**

**Section/topic # Checklist item Reported on page #**

**Table S2. Newcastle-Ottawa Scale quality assessment scores for the included studies.**
