# Supplementary material for: The Role of Circulating Tumor DNA in Advanced Non-Small Cell Lung Cancer Patients Treated With Immune Checkpoint Inhibitors: A Systematic Review and Meta-Analysis
Source: Front Oncol. 2021 Jul 21;11:671874. doi: 10.3389/fonc.2021.671874 (PMC8335591; doi:10.3389/fonc.2021.671874)
Supplement: Supplementary file 4 [file Table_2.docx]

Table S2. Newcastle-Ottawa Scale quality assessment scores for the included studies.

| **First Author** | **Selection** | **Comparability** | **Outcome** | **Total stars** |
| --- | --- | --- | --- | --- |
|  | (0-4) | (0-2) | (0-3) |  |
| Iijima, Y 2017 | ★★★ | ★★ | ★★★ | 8 |
| Gandara, D. R 2018 | ★★★★ | ★★ | ★★★ | 9 |
| Goldberg, S. B 2018 | ★★★ | ★★ | ★★★ | 8 |
| Raja, R 2018 | ★★★ | ★★ | ★★★ | 8 |
| Anagnostou, V 2019 | ★★★★ | ★★ | ★★★ | 9 |
| Guibert, N 2019 | ★★★ | ★★ | ★★★ | 8 |
| Chen, Y 2020 | ★★★ | ★★ | ★★★ | 8 |
| Jia, Q 2020 | ★★ | ★★ | ★★★ | 7 |
| Nabet, B. Y 2020 | ★★★★ | ★★ | ★★★ | 9 |
| Zulato, E 2020 | ★★★ | ★ | ★★★ | 7 |
